# A pragmatic, evidence-based approach to coding for abdominal wall reconstruction

**DOI:** 10.1007/s10029-021-02458-w

**Published:** 2021-10-30

**Authors:** Abdominal Core Health Quality Collaborative, B. K. Poulose, L.-C. Huang, S. Phillips, J. Greenberg, W. Hope, R. Janczyk, F. Malcher, A. Perez, R. A. Petersen, A. Prabhu, M. Reinhorn, J. A. Warren, N. White, M. J. Rosen

**Affiliations:** Abdominal Core Health Quality Collaborative, 4582 South Ulster Street, #201, Denver, CO 80237 USA

**Keywords:** Ventral hernia, Abdominal wall reconstruction, Current procedural terminology, Coding, Billing

## Abstract

**Purpose:**

Ambiguity exists defining abdominal wall reconstruction (AWR) and associated Current Procedural Terminology code usage in the context of ventral hernia repair (VHR), especially with recent adoption of laparoscopic and robotic-assisted AWR techniques. Current guidelines have not accounted for the spectrum of repair complexity and have relied on expert opinion. This study aimed to develop an evidence-based definition and coding algorithm for AWR based on myofascial releases performed.

**Methods:**

Three vignettes and associated outcomes were evaluated in adult patients who underwent elecive VHR with mesh between 2013 and 2020 in the Abdominal Core Health Quality Collaborative including: (1) no myofascial release (NR), (2) posterior rectus sheath myofascial release (PRS), and (3) PRS with transversus abdominis release or external oblique release (PRS-TA/EO). The primary outcome measure was operative time based on the following categories (min): 0–59, 60–119, 120–179, 180–239, and 240 + ; secondary outcomes included disease severity measures and 30-day postoperative outcomes.

**Results:**

15,246 patients were included: 7287(NR), 2425(PRS), and 5534(PRS-TA/EO). Operative time increased based on myofascial releases performed: 180–239 min (*p* < 0.05): NR(5%), PRS(23%), PRS-TA/EO(28%) and greater than 240 min (*p* < 0.05): NR (4%), PRS (17%), PRS-TA/EO(44%). A dose–response effect was observed for all secondary outcome measures indicative of three distinct levels of patient complexity and outcomes for each of the three vignettes.

**Conclusion:**

AWR is defined as VHR including myofascial release. Coding for AWR should reflect the actual effort used to manage these patients. We propose an evidence-based approach to AWR coding that focuses on myofascial release and is inclusive of minimally invasive techniques.

**Supplementary Information:**

The online version contains supplementary material available at 10.1007/s10029-021-02458-w.

## Introduction

Despite being a widely used term in ventral hernia repair (VHR), abdominal wall reconstruction (AWR) does not have a precise definition [1]. Significant advances have been made in AWR techniques over the past 15 years, most notably the development and dissemination of various myofascial releases [2–4]. When applied appropriately, these techniques can improve outcomes and reduce recurrence rates but are technically demanding to perform and often increase resource utilization [5, 6]. Recently, advances in both laparoscopic and robotic-assisted techniques have led to a growing array of AWR options incorporating the benefits of minimally invasive surgical (MIS) approaches [7–9].

The Abdominal Core Health Quality Collaborative (ACHQC) has been recognized as a United States (U.S.) Centers for Medicare and Medicaid Services (CMS) recognized Qualified Clinical Data Registry (QCDR) since 2017. Quality measures created by the ACHQC are submitted to CMS on behalf of requesting surgeons for the Merit-based Incentive Payment System (MIPS) [10]. While no standard definition of AWR exists, the ACHQC has led an effort to better characterize AWR in the context of surgical quality given the potential morbidity associated with these complex procedures. Through the QCDR system, CMS has recognized the ACHQC definition of AWR as VHR with myofascial release. Myofascial release, in turn, has been defined in the ACHQC as an abdominal wall fascial layer separated from a muscular layer since the inception of the collaborative in 2013 [11]. Despite this, adoption of this definition of AWR has not been widespread outside the ACHQC.

In addition, there is ambiguity regarding the appropriate Current Procedural Terminology (CPT) code usage for AWR techniques in the U.S. healthcare system. A significant advance in hernia coding guidance occurred in 2017 with the publication by Senkowski et al. in the Bulletin of the American College of Surgeons [12]. This document clarified many common scenarios encountered in both VHR and inguinal hernia repair. However, this effort failed to define appropriate coding for the spectrum of repair complexity in AWR and essentially excluded MIS techniques from AWR coding. In addition, since these recommendations were published, the ACHQC has gathered a vast amount of high-quality information on specific VHR and AWR techniques to guide coding efforts rather than relying on the voices of a few. The objective of this study was to determine whether three common clinical vignettes and associated outcomes can be used to develop an evidence-based definition and coding algorithm for AWR based on myofascial releases performed.

## Methods

### Design overview, data source, and hypothesis

A retrospective review of prospectively collected data was performed utilizing information from the ACHQC. The ACHQC is a national quality improvement initiative involving 450 surgeons in private practice or private practice with academic affiliation (51%) and academic practice (49%). Data are input during the preoperative, operative, and postoperative phases of care with a formal data assurance process for quality control as detailed previously [11]. Given that clarification of appropriate CPT coding use is facilitated through clinical vignettes and operative time, we developed three vignettes representative of common clinical situations in VHR based on the performance of myofascial release. We hypothesized that these three common clinical vignettes represent distinct patient groups with increasing operative time as the complexity of myfascial release is increased. The performance of this study was approved by The Ohio State University Wexner Medical Center Institutional Review Board and conformed to SQUIRE 2.0 recommendations.

### Population

Any adult patient (age 18 years or greater) undergoing elective ventral hernia repair with sublay mesh placement and completed 30-day follow-up between 2013 and 2020 in the ACHQC was eligible for the study.

### Clinical vignettes/comparison groups

**Fig. 1:**
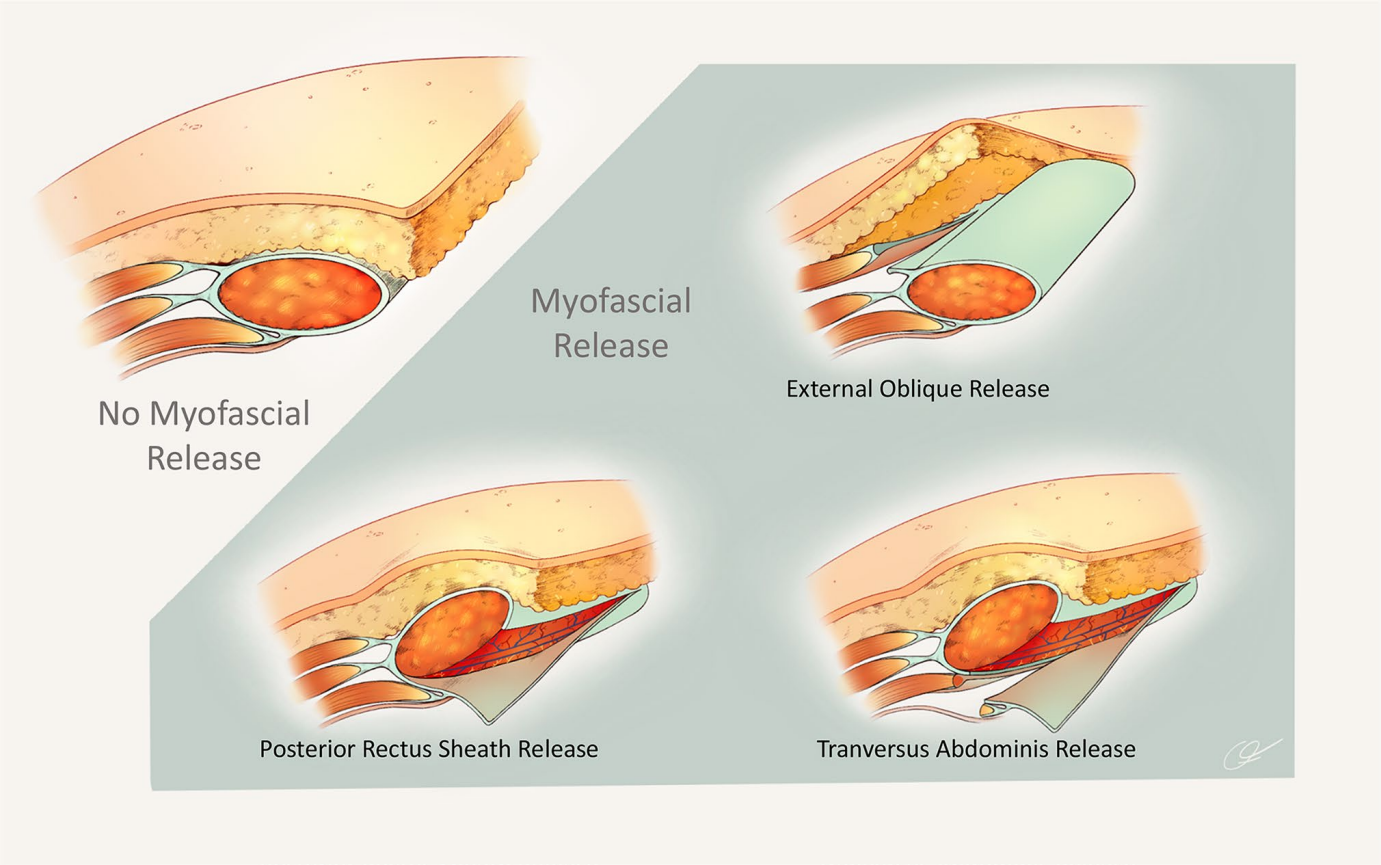
Common myofascial releases utilized for abdominal wall reconstruction—abdominal wall reconstruction is defined as ventral hernia repair that also includes the performance of myofascial release. Myofascial release is defined as an abdominal wall fascial layer separated from a muscular layer. Common types of myofascial releases are shown including posterior rectus sheath release, transversus abdominis release, and external oblique release. These releases can be performed using an open, laparoscopic, or robotic-assisted approach

Three common clinical scenarios utilized in VHR were chosen for evaluation: patients who underwent VHR with mesh and: (1) no myofascial release (NR) with intraperitoneal mesh placement, (2) posterior rectus sheath myofascial release (PRS) with retrorectus mesh placement, and (3) PRS with transversus abdominis release or external oblique release (PRS-TA/EO) with retromuscular mesh placement. These scenarios are summarized in Fig. 1.

### Primary outcome measure

The primary outcome measure was operative time as recorded in the ACHQC based on the following categories: 0–59, 60–119, 120–179, 180–239, and 240 + min. Operative time is a key factor in determining justification of more complex codes for operative interventions [13].

### Secondary outcome measures

Secondary outcome measures were chosen to reflect the spectrum of complexity in patient presentation, hernia disease severity, and resource utilization after repair. These include the proportion of patients undergoing recurrent hernia repair, transverse hernia width, length of hospital stay, any non-infectious postoperative complication at 30 days postop, surgical site infection at 30 days postop, and surgical site occurrence requiring procedural intervention at 30 days postop.

### Analysis

Categorical data are reported as proportions and comparisons made using Pearson’s Chi squared analysis. Continuous data are reported as median values with interquartile range with comparisons made using the Kruskal–Wallis test, accounting for three comparison groups. Statistical significance was achieved with a two tailed *p* value < 0.05. As the intent of the analysis was to determine if the three vignettes described distinct groups in terms of the primary and secondary outcome measures, no risk adjustment was performed between the groups. A double coding algorithm involving two separate analysts was utilized to identify the population and comparison groups, and to confirm the analysis.

## Results

We identified 15,246 patients meeting eligibility criteria with each vignette of: 7287 (NR), 2425 (PRS), and 5534 (PRS-TA/EO). Overall, the median age of patients was 59 years (interquartile range 49, 67) with 50% women and 86% White not of Hispanic origin. Demographics, comorbidities, and operative characteristics are shown in Table 1 and the Supplementary Table for each vignette. Patients had similar body mass indices across vignettes with higher rates of diabetes, hypertension, and chronic obstructive pulmonary disease in those undergoing either type of myofascial release (PRS or PRS-TA/EO). A smaller proportion of active smokers was noted in the most complex myofascial release group (PRS-TA/EO) reflective of patient selection for these techniques. Minimally invasive approaches (laparoscopic or robotic-assisted) were utilized more frequently in the NR group, while open approaches were utilized to a greater degree for vignettes including myfascial release (PRS or PRS-TA/EO). Notably for the more technically demanding MIS approaches, most were performed using a robotic-assisted or robotic-hybrid approach (25% PRS, 17% PRS-TA/EO) with a small proportion performed laparoscopically or via laparoscopic-hybrid approach (3% PRS, 1% PRS-TA/EO). Most hernias were M2–M4 in location, with an increased proportion of more complex locations (M1, M5, and L2, L3) and larger hernia widths found in the PRS-TA/EO group (Table 2).

**Table 1:** Operative characteristics by myofascial release performed^a^

	NR (7287)	PRS (*n* = 2425)	PRS-TA/EO (*n* = 5534)	*p* value
Incisional hernia (%)	59%	89%	97%	** < 0.001**
Centers for disease control wound class (%)				** < 0.001**
Class 1 (clean)	96%	86%	77%	
Class 2 (clean-contaminated)	3%	9%	13%	
Class 3 (contaminated)	1%	4%	9%	
Class 4 (dirty)	0%	1%	1%	
Operative approach (%)				** < 0.001**
Open	29%	71%	82%	
Laparoscopic	37%	1%	0%	
Robotic-assisted	28%	24%	12%	
MIS converted to open	1%	3%	1%	
Laparoscopic-hybrid	4%	0%	0%	
Robotic-hybrid	1%	1%	5%	
Mesh location (%)				** < 0.001**
Intraperitoneal	100%	0%	0%	
Retromuscular	0%	91%	54%	
Retromuscular and preperitoneal	0%	9%	46%	
Fascial closure achieved (%)	80%	99%	96%	** < 0.001**

The bold values indicate statistical significance

^a^*NR* (no myofascial release), *PRS* posterior rectus sheath myofascial release, *PRS-TA/EO* PRS with transversus abdominis release or external oblique release

**Table 2:** European hernia society (EHS) midline, lateral, and width classification of ventral hernias included in analysis

	NR (*n* = 7287)	PRS (*n* = 2425)	PRS-TA/EO (*n* = 5534)	*p* value
EHS classification—midline				***p* < 0.001**
M1—subxiphoidal	5%	12%	29%	
M2—epigastric	35%	66%	77%	
M3—umbilical	69%	83%	86%	
M4—infraumbilical	14%	48%	70%	
M5—suprapubic	4%	14%	25%	
No midline component	7%	3%	5%	
EHS classification—lateral				***p* < 0.001**
L1—subcostal	3%	1%	6%	
L2—flank	6%	4%	14%	
L3—iliac	4%	2%	11%	
L4—lumbar	0%	0%	2%	
No lateral component	88%	94%	74%	
EHS classification—width				***p* < 0.001**
W1— < 4 cm	59%	12%	1%	
W2— ≥ 4–10 cm	35%	68%	24%	
W3— ≥ 10 cm	6%	20%	75%	

The bold values indicate statistical significance

**Fig. 2:**
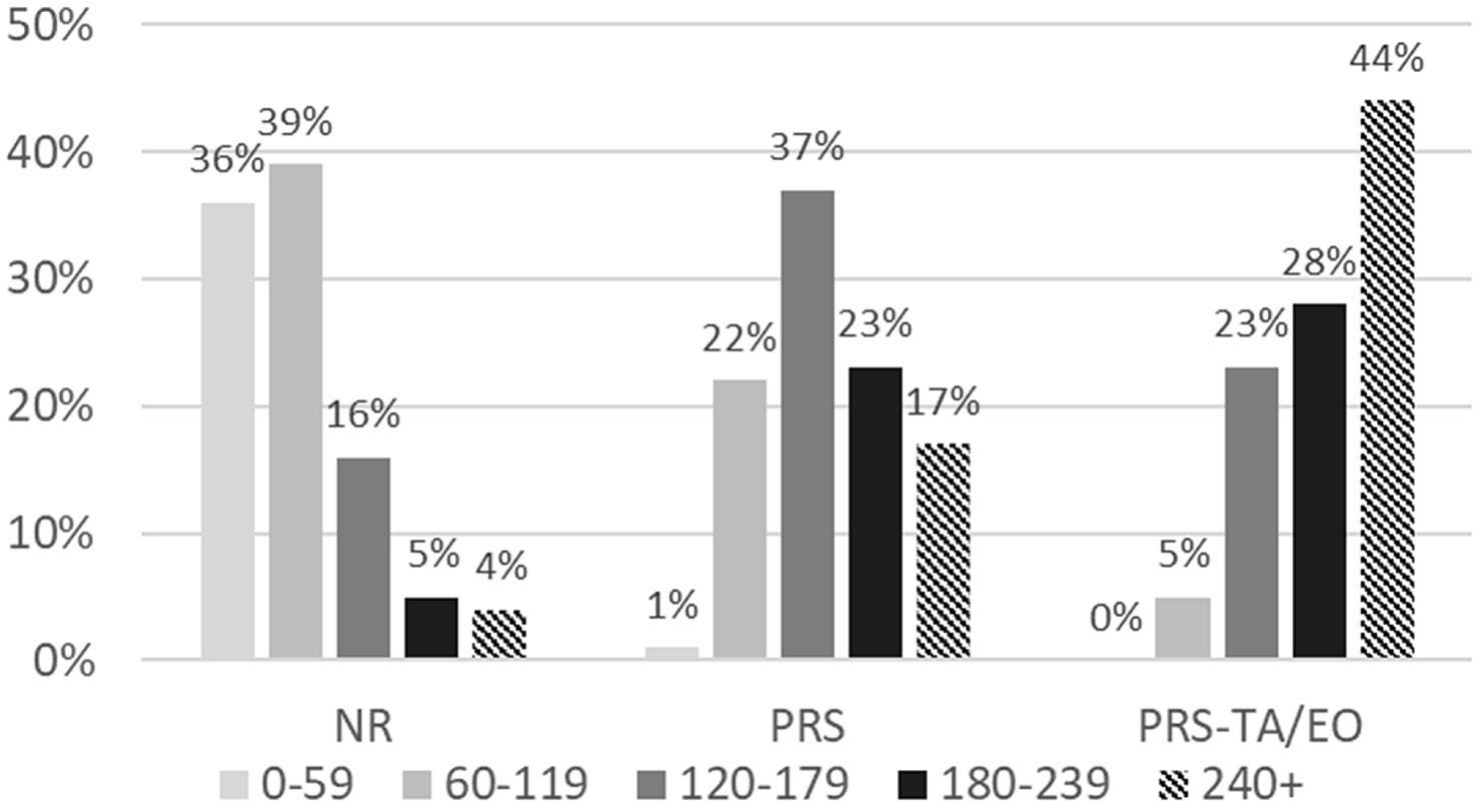
Operative time by myofascial release performed—increasing operative times are observed progressing from no release (NR) to increasingly more complex myofascial releases including posterior rectus sheath myofascial release (PRS), and PRS with transversus abdominis release or external oblique release (PRS-TA/EO)

Results for the primary outcome measure, operative time, are shown in Fig. 2. Progressing from NR to increasingly more complex myofascial releases (PRS then PRS-TA/EO), increasing operative distribution times are noted. The NR vignette had the shortest operative time with most operations (39%) taking less than 60–119 min. For patients undergoing PRS, the majority of patients underwent operation in an intermediate time frame of 120–179 min (36%). Patients in the PRS-TA/EO vignette had the longest operative time with most (44%) undergoing procedures 240 min or longer. Of these patients, 95% underwent release of the TA, 4% underwent release of the EO, and 1% underwent releases of both the TA/EO. Two subgroup analyses were performed separating patients undergoing repair of incisional hernia (78%, *n* = 11,852) and primary ventral hernia (22%, *n* = 3,394, e.g., umbilical hernia and epigastric hernia) with similar results as the combined ventral hernia analysis (Table 3).

**Table 3:** Subgroup analysis for operative time: incisional hernia and primary ventral hernia

	NR (*n* = 7287)	PRS (*n* = 2425)	PRS-TA/EO (*n* = 5534)	*p* value
Operative time (min)				
Incisional hernia	(*n* = 4320)	(*n* = 2159)	(*n* = 5373)	***p* < 0.001**
0–59	23%	1%	0%	
60–119	44%	21%	5%	
120–179	22%	36%	23%	
180–239	7%	24%	28%	
240 +	5%	17%	44%	
Primary ventral hernia	(*n* = 2967)	(*n* = 266)	(*n* = 161)	***p* < 0.001**
0–59	56%	2%	0%	
60–119	32%	32%	7%	
120–179	8%	37%	24%	
180–239	2%	15%	24%	
240 +	1%	14%	45%	

The bold values indicate statistical significance

Secondary outcome measure results are detailed in Table 4. For each secondary outcome including the proportion of patients undergoing recurrent hernia repair, transverse hernia width, length of hospital stay, any non-infectious postoperative complication, surgical site infection, and surgical site occurrence requiring procedural intervention, a ‘dose response’ effect was observed with NR being the least complex, PRS having intermediate complexity, and PRS-TA/EO having the highest complexity.

**Table 4:** Secondary outcome measures by myofascial release performed^a^

	NR (7287)	PRS (*n* = 2425)	PRS-TA/EO (*n* = 5534)	*p* value
Patients undergoing recurrent repair (%)	21%	37%	50%	** < 0.001**
Transverse hernia width (cm, median (interquartile range))	3 (2, 5)	6 (5, 9)	13 (9, 16)	** < 0.001**
Length of hospital stay (days, median (interquartile range))	0 (0, 1)	3 (1, 4)	5 (3, 7)	** < 0.001**
Any non-infectious complication at 30 days (%)	12%	21%	29%	** < 0.001**
Surgical site infection at 30 days (%)	1%	3%	6%	** < 0.001**
Surgical site occurrence requiring procedural intervention at 30 days (%)	2%	4%	8%	** < 0.001**

The bold values indicate statistical significance

^a^*NR* no myofascial release, *PRS* posterior rectus sheath myofascial release, *PRS-TA/EO* PRS with transversus abdominis release or external oblique release

## Discussion

In this study, we have identified three distinct clinical groups based on common vignettes encountered in practice. This approach utilizes the definition of AWR as VHR with myofascial release. Myofascial release, in turn, is defined as an abdominal wall fascial layer separated from a muscular layer. Patients undergoing VHR alone without myofascial releases would not be considered having undergone AWR. Patients undergoing VHR with either PRS, PRS-TA/EO or other forms of myofascial release, would be characterized as having undergone AWR.

One of the key strengths of the ACHQC is the ability to gather detailed operative information across multiple practices that can be used for a wide range of high impact efforts. For this study, detailed information regarding myofascial release was correlated with operative time to characterize three common clinical vignettes. Using this approach, we were able to show that these scenarios represent distinct VHR groups, and that these groups represent a step-wise array of operative technical complexity as reflected by operative time. In addition, these groups comprise separate patients populations that warrant increasingly complex management when considering the utilization of myofascial release. This type of analysis would not be possible without detailed and granular operative information across a wide range of institutions, surgeons, and patients.

**Fig. 3:**
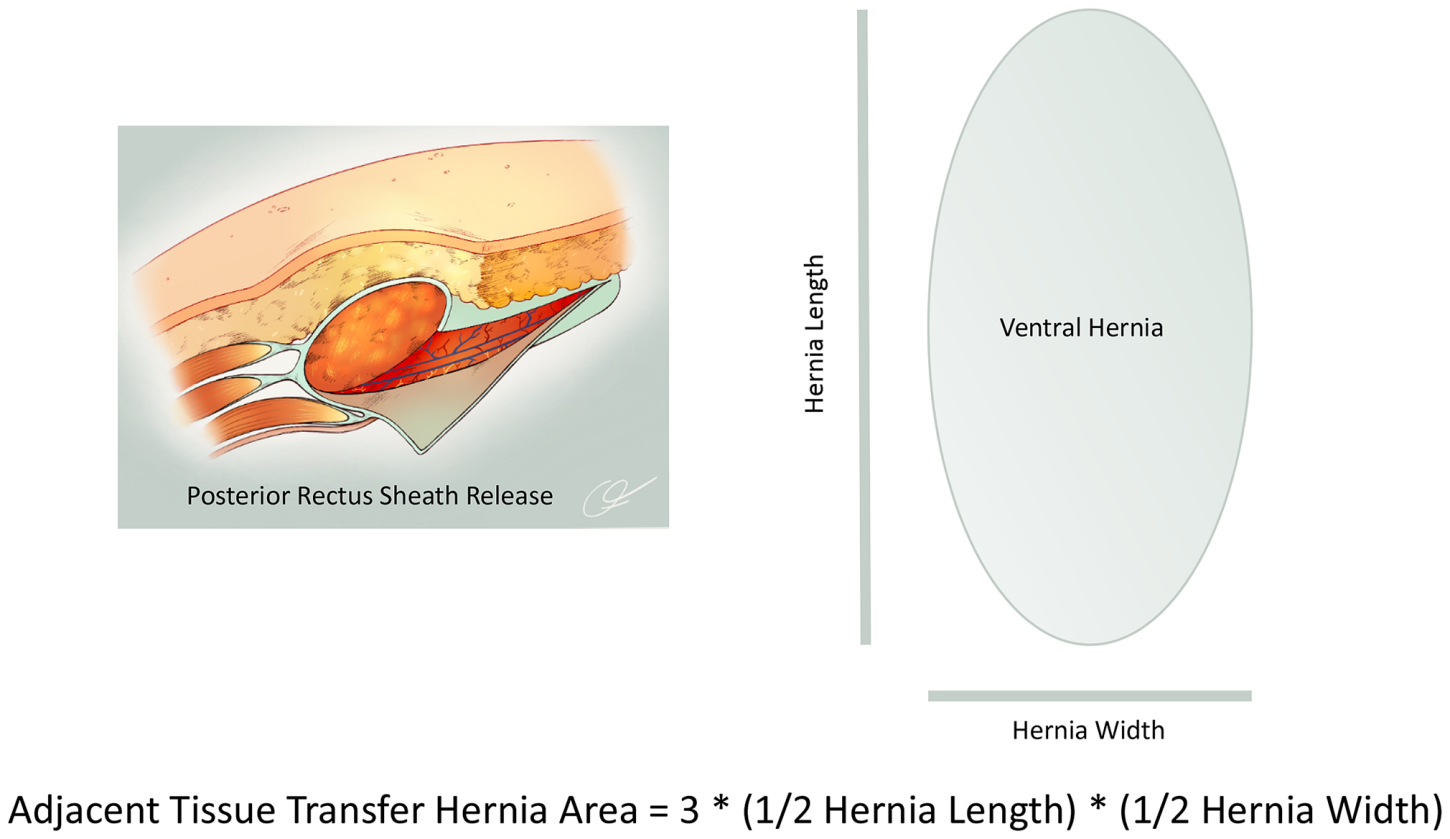
Adjacent tissue transfer area calculation for ellipse-shaped ventral hernia—to calculate and document the adjacent tissue transfer area (CPT 14301 and 14302) for most ventral hernias, hernia area is calculated based on the area of an ellipse (area = π*radius A*radius B). For example, if the hernia length is 10 cm and width is 6 cm, the area reported for posterior rectus sheath myofascial release (adjacent tissue transfer) billing would be (3*5 cm*3 cm) = 45 square centimeters. CPT 14301 is reported for any defect 30.1 square centimeters to 60.0 square centimeters and CPT 14302 is reported for each additional 30.0 square centimeters or part thereof. These codes should not be used for ventral hernias with area of 30.0 square centimeters or less, even if posterior rectus sheath myofascial release is utilized.

In the United States, the current recommendations as published by ACS Bulletin guidelines stipulate that CPT code 15734 (muscle, myocutaneous or fasciocutaneous flap, trunk) is reserved for myofascial releases other than PRS [12]. This would include PRS-TA/EO. Per these recommendations, PRS (such as the Rives-Stoppa repair without additional releases) is billed similarly to that of VHR without releases (NR). Our data support the use of an intermediate CPT code for PRS that reflects the additional technical expertise, operative time, and patient complexity when PRS is utilized but not to the level of CPT 15734. For PRS, we propose utilization of the adjacent tissue transfer code CPT 14301 (adjacent tissue transfer or rearrangement, any area, defect 30.1 square centimeters to 60.0 square centimeters), and if needed, 14302 (each additional 30.0 square centimeters or part thereof) to represent the additional work performed when PRS is utilized [14]. The CPT 2021 coding manual specifically indicates that ‘undermining alone of adjacent tissues to achieve closure, without additional incisions, does not constitute adjacent tissue transfer.’[14] As such, raising subcutaneous flaps alone does not justify utilization of this code. However, PRS does involve an additional incision in the posterior rectus sheath which then allows medialization of the rectus complex to facilitate fascial closure and VHR. This would also be considered a type of AWR as a myofascial release (PRS) is performed. There is no associated bilateral modifier for codes 14301 or 14,02; it is necessary for the surgeon to document the total area involved in tissue transfer. Since the vast majority of ventral hernias are elliptical in shape, we suggest utilization of the formula for an ellipse (area = π*radius A*radius B) to accurately document the square centimeter area for adjacent tissue transfer as appropriate. The ACHQC has adopted the European Hernia Society method of reporting ventral hernia dimensions using length and width [15]. A practical formula that can be used to report area is: hernia area = 3*(1/2 hernia length)*(1/2 hernia width). Documentation of hernia length and width should be provided in every VHR or AWR operative report. For example, if the hernia length is 10 cm and width is 6 cm, the area reported for PRS billing would be (3*5 cm*3 cm) = 45 square centimeters (Fig. 3). CPT 14301 should not be applied to repair of defects 30 square centimeters or less in area, even if PRS was utilized. Note that in addition to use of CPT 14301/14302 in this scenario, any codes reflective of concomitant work should also be reported (e.g., VHR and mesh removal) [12, 16].

When additional myofascial releases are utilized beyond PRS (such as PRS-TA/EO), we do not recommend reporting both CPT 14301/14302 and CPT 15734. When performing TA release, PRS is inherent to the procedure and is captured in a single instance of CPT 15734. Similarly, when performing EO release, PRS may or may not be utilized. A bilateral modifier ( – 59) is allowed for CPT 15734 and is added to the second (contralateral) instance of CPT 15734. No more than two instances of CPT 15734 should be reported for a given AWR operation. When additional myofascial releases are utilized beyond PRS (such as PRS-TA/EO), we do recommend reporting any codes reflective of concomitant work (e.g., VHR and mesh removal) as per previous published recommendations [12, 16]. The overall recommendations are summarized in Table 3.

The performance of AWR should be reserved for clinical situations warranting utilization (and billing) of advanced myofascial release techniques. Although determing the appropriateness of AWR is beyond the scope of this study, each surgeon is urged to use their best judgment based on their own skillset and the clinical needs of each patient. Data have consistently shown that as the time interval after VHR increases, the risk of recurrence also increases [17, 18]. This suggests that ventral hernia is a chronic disease and for a given patient, an array of surgical interventions may be needed over time to maintain quality of life. Premature utilization of AWR techniques, when not necessary, hampers the care of patients over time.

The utilization of MIS techniques (laparoscopic or robotic-assisted VHR and AWR) should not preclude the use of AWR codes. These MIS techniques can be more technically demanding and require equivalent time to their open counterparts; the work performed should be appropriately reflected in professional coding and billing [8, 9]. Also, not allowing AWR codes for use in MIS would create a disincentive for utilization of these approaches that may offer clinical benefit.

The results of this study should be interpreted in light of several limitations. Data from the ACHQC may not be representative of all patients undergoing hernia repair in the United States. In spite of this, the relative outcome differences seen between clinical vignettes should also be reflected in a non-ACHQC population. As the primary outcome measure was operative time, the analysis did not consider other components of an operation that may contribute to extended operative time besides the performance of myofascial release (e.g., adhesiolysis). A patient in the NR group may have had a prolonged operative time due to these issues; conversely, a patient in the PRS-TA/EO group may have had an expeditious procedure without adhesiolysis. Similarly, patients with larger hernia sac volumes relative to the transverse hernia width itself (e.g., loss of domain) was a characteristic not accounted for in this analysis. The data averaged over thousands of patients in this study though do reveal the relationship between increased myofascial release complexity and increased operative time. Adjuncts to AWR such as botulinum toxin A use and preoperative pneumoperitoneum were not captured in this analysis. No attempt was made to adjust for differences between the three clinical vignettes and resulting comparison groups. This was intentional as we wished to highlight the clinical differences between these groups as opposed to control for several variables and isolate a single factor for comparison. The coding scheme recommended may not be agreeable to all who have traditionally used these codes. We recognize this as an issue and attempted to provide an evidence-based approach to proper AWR coding where much ambuigity has existed. Our professional coding colleagues rely on Societies to provide guidance on appropriate use of CPT codes reflective of different scenarios.

As the coding recommendations in this study pertain largely to the United States healthcare system and other systems utilizing CPT coding, its use in other systems may be limited. However, the concept of defining AWR as VHR with myofascial release, and in turn, myofascial release defined as an abdominal wall fascial layer separated from a muscular layer transcends international boundaries and health systems. This precise definition of AWR can be utilized in any health system to differentiate these procedures from non-AWR VHR.

In conclusion, AWR should be defined as VHR including appropriate myofascial release(s) performed. Distinct professional billing codes for ventral hernia repair without a release, PRS, and PRS-TA/EO should be utilized to reflect appropriate patient complexity, operative time, and outcomes regardless of the operative approach utilized (open, laparoscopic, or robotic-assisted).

## Supplementary Information

Below is the link to the electronic supplementary material.

Supplementary file1 (DOCX 25 KB)

## Data Availability

Available upon request and approval through the ACHQC.

## Code availability

Available upon request and approval through the ACHQC.
