# Supplementary material for: A pragmatic, evidence-based approach to coding for abdominal wall reconstruction
Source: Hernia. 2021 Oct 30;26(2):589–97. doi: 10.1007/s10029-021-02458-w (PMC9012717; doi:10.1007/s10029-021-02458-w)
Supplement: Supplementary file 1 — Supplementary file1 (DOCX 25 KB) [file 10029_2021_2458_MOESM1_ESM.docx]

Supplemental Table – Demographics and Comorbidities by Myofascial Release Performed^a^

|  | NR (7,287) | PRS  (n=2,425) | PRS-TA/EO  (n=5,534) | p value |
| --- | --- | --- | --- | --- |
| Demographics |  |  |  |  |
| Age (years, median (interquartile range)) | 57 (47, 67) | 58 (48, 67) | 60 (51, 68) | **<0.001** |
| Women (%) | 47% | 54% | 52% | **<0.001** |
| White not Hispanic (%) | 84% | 85% | 89% | **<0.001** |
| Body Mass Index (kg/m^2^, median (interquartile range)) | 32 (28, 36) | 32 (28, 36) | 32 (28, 36) | 0.08 |
| Comorbidities |  |  |  |  |
| Current Nicotine Use (%) | 12% | 11% | 9% | **<0.001** |
| Diabetes (%) | 17% | 21% | 22% | **<0.001** |
| COPD (%) | 6% | 6% | 8% | **<0.001** |
| Hypertension (%) | 47% | 53% | 56% | **<0.001** |
| Modified Hernia Grade  1 | 29% | 24% | 16% | **<0.001** |
| 2 | 67% | 62% | 61% |  |
| 3 | 4% | 14% | 23% |  |
| ASA Class  1  2  3  4 | 8%  49%  41%  2% | 3%  41%  53%  2% | 1%  27%  69%  3% | **<0.001** |

^a^NR (no myofascial release), PRS (posterior rectus sheath myofascial release), PRS-

TA/EO (PRS with transversus abdominis release or external oblique release)
